# MMSET dysregulates gene expression in myeloma through global and focal changes in H3K36 and H3K27 methylation

**DOI:** 10.1186/1756-8935-6-S1-P66

**Published:** 2013-03-18

**Authors:** Relja Popovic, Eva Martinez-Garcia, Quanwei Zhang, Teresa Ezponda, Mrinal Y Shah, Yupeng Zhang, Yanwen Jiang, Christine M Will, Eliza C Small, Marinka Bulic, Olivier Elemento, Neil Kelleher, William Kath, Ji-Ping Wang, Jonathan D Licht

**Affiliations:** 1 Division of Hematology/Oncology, Northwestern University Feinberg School of Medicine, Chicago, Illinois, USA; 2 Department of Statistics, Northwestern University, Evanston, Illinois, USA; 3 Department of Chemistry and Molecular Biosciences, Chemistry of Life Processes Institute, Northwestern University, Evanston, Illinois, USA; 4 HRH Prince Alwaleed Bin Talal Bin Abdulaziz Alsaud Institute for Computational Biomedicine and Department of Physiology and Biophysics, Weill Cornell Medical College, New York, New York, USA; 5 Department of Engineering Sciences and Applied Math, Northwestern University, Evanston, Illinois, USA

## 

The MMSET (Multiple Myeloma SET domain) protein is overexpressed in multiple myeloma patients harboring the translocation t(4;14), and is believed to be the driving factor in the pathogenesis of this subtype of myeloma. Our studies showed that overexpression of MMSET in myeloma cells induces a global increase in H3K36 dimethylation, a mark associated with actively transcribed genes, and a concomitant genome-wide loss of H3K27 trimethylation. This effect is due to an enhanced rate of H3K36 methylation as well as an increased rate of H3K27 demethylation leading to altered gene expression. Using ChIP-Seq and genetically matched cells in which the overexpressed MMSET allele was knocked out (KMS11-TKO), we found that increased H3K36 methylation due to MMSET disrupts the normal genomic H3K36me2 architecture, from a mostly promoter-enriched mark to a modification spread throughout the genome. Additionally, many loci activated in response to MMSET showed the loss of H3K27 methylation near the transcriptional start site. Surprisingly, while MMSET-overexpressing cells lose the H3K27me3 mark globally, specific loci display enrichment of this modification and are transcriptionally repressed. This is associated with a shift in the genomic localization of the H3K27 methyltransferase, EZH2, in the presence of MMSET. Thus, in addition to gene activation through increased H3K36 methylation, overexpression of MMSET can also induce gene repression by imposing EZH2 and H3K27me3 accumulation at specific genomic loci.

Using KMS11-TKO cells, we performed a structure-function study to identify important domains of MMSET. Re-introduction of the wild-type MMSET into the TKO cells induces the H3K36/H3K27 epigenetic switch, enhances proliferation and colony formation of these cells, and leads to differential gene expression. These biological activities require all four PHD finger domains, the second PWWP domain and the functional SET domain. When MMSET constructs containing mutations or deletions in one of these domains were overexpressed in TKO cells, the resulting protein failed to methylate histones, alter growth or change gene expression. Furthermore, point mutations in the PHD domains 2 or 3 prevented MMSET from binding to chromatin and altering histone methylation. By contrast, an MMSET construct lacking the C-terminal PHD4 domain bound chromatin, induced methylation of H3K36, but was unable to mediate a complete loss of the H3K27me3 mark or fully stimulate growth. Together this suggests that pathogenic activity of MMSET could be interrupted by blocking its binding to chromatin, its intrinsic H3K36 methylation activity or by blocking demethylases of H3K27 that might interact with MMSET.

To validate MMSET as a therapeutic target, we used KMS11 t(4;14)+ cells that express *MMSET* specific shRNA in the presence of doxycycline. KMS11 cells rapidly formed tumors when injected into immunocompromised mice. Doxycycline treatment caused a dramatic decrease in the volume of established tumors, extended survival in mice and, in some cases, completely eliminated the tumor burden. Together, our work elucidates some of the mechanisms used by MMSET to induce an oncogenic phenotype and identifies domains to be considered in designing novel inhibitors of MMSET function.

